# Successful Application of Adaptive Emotion Regulation Skills Predicts the Subsequent Reduction of Depressive Symptom Severity but neither the Reduction of Anxiety nor the Reduction of General Distress during the Treatment of Major Depressive Disorder

**DOI:** 10.1371/journal.pone.0108288

**Published:** 2014-10-20

**Authors:** Carolin M. Wirtz, Anna Radkovsky, David D. Ebert, Matthias Berking

**Affiliations:** 1 Department of Clinical Psychology and Psychotherapy, University of Marburg, Marburg, Hesse, Germany; 2 Department of Clinical Psychology and Psychotherapy, University of Erlangen- Nuremberg, Erlangen, Baveria, Germany; 3 Division for Clinical Psychology and Psychotherapy, Department for Psychology, Leuphana University Lueneburg, Lueneburg, Lower Saxony, Germany; University of Granada, Spain

## Abstract

**Objective:**

Deficits in general emotion regulation (ER) skills have been linked to symptoms of depression and are thus considered a promising target in the treatment of Major depressive disorder (MDD). However, at this point, the extent to which such skills are relevant for coping with depression and whether they should instead be considered a transdiagnostic factor remain unclear. Therefore, the present study aimed to investigate whether successful ER skills application is associated with changes in depressive symptom severity (DSS), anxiety symptom severity (ASS), and general distress severity (GDS) over the course of treatment for MDD.

**Methods:**

Successful ER skills application, DSS, ASS, and GDS were assessed four times during the first three weeks of treatment in 175 inpatients who met the criteria for MDD. We computed Pearson correlations to test whether successful ER skills application and the three indicators of psychopathology are cross-sectionally associated. We then performed latent growth curve modelling to test whether changes in successful ER skills application are negatively associated with a reduction of DSS, ASS, or GDS. Finally, we utilized latent change score models to examine whether successful ER skills application predicts subsequent reduction of DSS, ASS, or GDS.

**Results:**

Successful ER skills application was cross-sectionally associated with lower levels of DSS, ASS, and GDS at all points of assessment. An increase in successful skills application during treatment was associated with a decrease in DSS and GDS but not ASS. Finally, successful ER skills application predicted changes in subsequent DSS but neither changes in ASS nor changes in GDS.

**Conclusions:**

Although general ER skills might be relevant for a broad range of psychopathological symptoms, they might be particularly important for the maintenance and treatment of depressive symptoms.

## Introduction

Major depressive disorder (MDD) is one of the most common mental disorders [1] and is considered to be a leading cause of disease burden worldwide [2]. Despite the availability of effective pharmacological and psychological treatments [3], outcome research also indicates that even when treated with evidence-based interventions, MDD remains a highly prevalent [1], usually recurrent [4], and potentially chronic problem [5], [6]. In patients suffering from MDD, anxiety, nervousness, and their somatic correlates are common comorbid symptoms. For example, Melartin and colleagues [7] found that nearly 60% of patients with an episode of MDD suffered from at least one comorbid anxiety disorder. The most frequent comorbid anxiety disorder in patients with MDD is social phobia, followed by generalized anxiety disorder, and panic disorder [8], [9]. MDD with high levels of anxiety symptoms has been found to be associated with an even greater functional impairment, chronicity [10], delayed response to treatment [11], more severe depression, and an increased risk of suicidality [12]. Moreover, the presence of a comorbid anxiety disorder predicts a poorer long-term outcome and a greater familial prevalence of MDD [11], [13]. These findings indicate that more research is needed to improve the efficacy and sustainability of psychotherapeutic treatments for MDD especially when co-occurring anxiety disorders have to be taken into account. Such research should include studies aiming to identify mechanisms that facilitate change in evidence-based treatments for depression and anxiety symptoms [14], [15]. Potentially relevant mechanisms include transdiagnostic factors, which can be hypothesized to be relevant for either common symptoms co-occurring in depressive and anxiety disorders or common causes of these two forms of psychopathology disorders. With regard to common symptoms, several studies have shown that depression and anxiety disorders share a general distress factor [16]–[18].

Recent research has focused on general deficits in emotion regulation (ER) as a putative factor in the maintenance of various forms of psychopathology [19]–[23]. ER refers to the set of processes whereby people seek to monitor, evaluate, and redirect the spontaneous flow of their emotions to accomplish their needs and goals [24]–[26]. Deficits in general ER skills may contribute to the development of both MDD and anxiety disorders in several ways. First, the inability to down-regulate undesired affective states may lead to an escalation or perpetuation of each of these states and may thus enhance an individual’s likelihood of meeting the diagnostic criteria for anxiety disorders or depression [27], [28]. Second, deficits in ER skills may lead to increased aversive emotional experiences in general and may thus increase an individual’s vulnerability to developing mental disorders in general [21], [23]. More specifically, the absence of effective ER skills likely amplifies the risk that an individual’s evaluates the current situation as aversive and beyond one’s control, which is a relevant antecedent of anxiogenic and depressogenic information processing [29], [30]. Finally, in the absence of effective ER skills, the interaction of subthreshold symptoms of anxiety and depression may significantly impede an individual’s attempt to cope with both anxiety and depression. For example, (subthreshold) anxiety may interfere with the individual’s attempt to engage in positive activities when working to overcome depression; similarly, feelings of dysphoria, helplessness, and hopelessness may interfere with the individual’s attempt to confront feared stimuli in order to overcome anxiety [31], [32].

Based on the assumption that deficits in ER contribute to the development and maintenance of various mental disorders, Berking [33]–[35] proposed a skill-based model of adaptive coping with emotions (ACE) that facilitates the use of the notoriously broad and abstract concept of ER for clinical purposes. The ACE model conceptualizes adaptive ER as a situation-dependent interaction between the following ER skills: (1) the ability to be consciously aware of emotions, (2) the ability to identify emotions, (3) the ability to correctly label emotions, (4) the ability to identify what has caused and what maintains one’s present emotions, (5) the ability to actively modify emotions in an adaptive manner, (6) the ability to accept undesired emotions when they cannot be changed, (7) the ability to tolerate undesired emotions when they cannot be changed, (8) the ability to approach and confront situations that are likely to trigger negative emotions if this is necessary to attain personally relevant goals, and (9) the ability to provide compassionate self-support when working to cope with challenging emotions. The model additionally includes the hypothesis that only the acceptance/tolerance and modification of undesired emotions are ultimately relevant for mental health. All other skills are only necessary to the extent that they facilitate an individual’s ability to successfully accept/tolerate or modify undesired emotions.

Numerous studies have provided empirical evidence on the relevance of skills included in the ACE model for both depression and anxiety disorders. For example, cross-sectional studies suggest that symptom severity in both MDD and anxiety disorders are associated with difficulties in identifying and labeling emotions [36]–[39], accepting and tolerating negative emotions [40]–[47], compassionately supporting oneself when facing challenging emotions [48]–[50], and adaptively modifying emotions [51]–[53]. Moreover, in both disorders, patients report experiencing high levels of shame and hopelessness [54], [55] and using dysfunctional ER strategies when working to cope with challenging experiences such as rumination or avoidance [19], [56].

Longitudinal studies suggest that deficits in ER predict the subsequent severity of depression and anxiety symptoms in clinical and nonclinical samples. For example, in nonclinical samples, deficits in ER skills application negatively predicted subsequent anxiety symptom severity over a 2-week period [33] and the level of both depression and anxiety over a 5-year interval [57], [58]. Additional longitudinal evidence is provided by studies showing that rumination in response to undesired emotions prospectively predicted the level of depressive symptoms as well as the prevalence of anxiety disorders in clinical and nonclinical samples as well as adults, adolescents, and children [59]–[61]. Studies that have failed to find evidence for the importance of general ER skills for both depression and anxiety include a study by McLaughlin and colleagues [32], in which ER also predicted subsequent increases in anxiety symptoms but did not predict the level of depressive symptoms in a large sample of adolescents over a 7-month period. Similarly, in the aforementioned study by Berking [33], ER skills significantly predicted anxiety symptoms and negative affect, but a nonsignificant trend was found for the ability of ER skills to predict subsequent depression. However, in another study, ER skills negatively predicted the level of depressive symptoms over a 5-year interval in a nonclinical sample [58]. Therefore, Berking and colleagues argued that general ER skills are relevant first for preventing the onset of anxiety and negative affect and subsequently for preventing the onset of depressive symptoms. However, at this point, this hypothesis has not yet been systematically investigated.

In addition to clarifying moderators explaining the partly inconsistent findings regarding the transdiagnostic relevance of ER skills, the present study investigates the reciprocal associations between general ER skills and symptoms of depression, anxiety, and general distress over the course of treatment, as studies on these associations are lacking in the literature to this point. In particular, a general distress factor has been included in the analyses in the present study because it has frequently been associated with depressive and anxiety disorders. Distress may simply be a symptom occurring in both forms of psychopathology, or it may even be the common cause for the onset of depressive and anxiety disorders. If fostering ER skills reduces the intensity of symptoms of these three domains, adopting such a transdiagnostic approach might be more economical than compiling a number of strategies that each focus on one domain of psychopathology [20].

The present study thus aimed to clarify the reciprocal associations between the successful application of arguably adaptive general ER skills (as included in the ACE model) and depressive symptom severity (DSS), anxiety symptom severity (ASS), and general distress symptom severity (GDS) over the course of treatment for MDD. More specifically, we first tested whether the ability to successfully apply adaptive ER skills would be cross-sectionally associated with lower levels of DSS, ASS, or GDS during four stages of treatment for MDD. Second, we tested whether changes in successful ER skills application would be negatively associated with changes in DSS, ASS, or GDS during treatment for MDD. Finally, we aimed to clarify whether successful ER skills application would predict subsequent changes in DSS, ASS, and GDS during treatment for MDD.

## Methods

### Procedures and Participants

The study was conducted in a German mental health hospital between August 2010 and August 2012 [62]. Once a week, participants completed a set of self-report questionnaires that assessed successful ER skills application, depressive symptom severity (DSS), anxiety symptom severity (ASS), and general psychological distress severity (GDS) (see Measures). Given that the majority of patients who were treated in the hospital stayed for at least three weeks, we chose to cover this 3-week period in the analyses. The assessments took place on a weekly basis, starting in the first week of hospital admission. If participants missed an assessment, they were allowed to make up the assessment within the next two days. All participants were invited to complete the four points of assessment, which were provided through an online assessment tool. Data entry, transmission, and storage were strictly protected from unauthorized access. All study procedures followed internationally accepted human research guidelines, such as the Helsinki Protocol, and received approval by the ethics committee of Marburg University. All participants provided their written consent to participate in the study. The written consent has been approved by the ethics committee.

In order to be eligible for the study, participants were required to meet the following criteria: (a) current diagnosis of MDD according to the DSM-IV criteria, (b) pre-treatment BDI score of 11 or above, (c) 18 years of age or above, (d) sufficient German language skills to complete the questionnaires, and (e) no current alcohol or drug addiction, psychoses, bipolar disorder, brain damage, or other severe somatic disorders requiring other treatments. Based on sensitivity and specificity analyses, Riedel and colleagues [63] recently recommended 11 or above as a clinical cut-off score for the German version of the BDI, which is used in the present study. To maximize the external validity of the findings, no further exclusion criteria were considered (e.g., regarding comorbidity, antidepressant medication, or suicidal tendencies).

Within the first week of treatment, diagnostic assessments were conducted. Participants were interviewed with the Structured Clinical Interview for the DSM-IV (German version: SCID [64]). All raters who conducted the diagnostic interviews had a Bachelor’s degree or higher in clinical psychology and had all received extensive training in the SCID interview (18 hours of training by a certified trainer). In addition, all raters were supervised by experienced psychotherapists (either psychologists or physicians with a Master’s degree or higher in psychology or medicine).

The final sample consisted of 175 participants. The average total length of treatment was about seven weeks (*M* = 7.2; *SD* = 2.35; *range* = 2.85–22.89). The majority of the participants had at least one comorbid Axis I diagnosis (58.9%). The most common comorbidity included anxiety disorders (any anxiety disorder: 51.4%; social phobia: 26.1%; agoraphobia: 20.7%; generalized anxiety disorder: 18%; panic disorder: 14.4%; posttraumatic stress disorder: 10.8%), followed by comorbid somatoform disorders (35.1%) and dysthymia (13.5%). About one-third of the participants (36%) met the criteria for at least two comorbid Axis I disorders. About one-quarter of the participants (23.0%) met the criteria for at least one Axis II disorder. Among these disorders, the most common were avoidant personality disorder (8.6%), obsessive-compulsive personality disorder (3.9%), and borderline personality disorder (3.3%).

All of the participants were Caucasian (which is quite representative of the German population), the majority were women (57.7%), and the average age was 46.7 years (*SD* = 10.8, *range* = 18–71). The highest level of education (“Abitur”) was reported by 40% of the sample, 35.4% reported to have completed the second highest level of education (“Realschulabschluss”), and 15.4% had completed the lowest level of education. Nearly half of the participants (44.6%) were married, 13.1% were divorced, and 28% had never been married; moreover, 59.4% had at least one child.

### Treatment

During the treatment period under investigation, the participants received an average of 3.63 hours (*SD* = 1.29, *range* = 3.00–6.00) of individual and 23.64 hours (*SD* = 1.58, *range* = 16.00–26.00) of group psychotherapy. About half of the group-based therapy specifically focused on depression, utilizing cognitive behavioral therapy techniques developed and validated for this disorder [65]. All of the participants received group treatment for depression during the first three weeks (followed by group therapy for comorbid disorders, such as anxiety disorders, if they were present). Psychotherapeutic interventions were supplemented with sports and arts therapy as well as medical treatment when necessary. All treatments were based on a cognitive behavioral rationale and included techniques such as behavior analyses, behavioral activation, cognitive restructuring, and relaxation training [66]. Psychotherapeutic treatment was delivered by experienced therapists and therapists in training-all of whom had a Master’s degree in psychology or medicine. Supplementary treatments were delivered by trained nurses, sports and art therapists, physiotherapists, and medical doctors. Treatment integrity was ensured through regular team meetings and weekly supervision by licensed senior therapists. Treatment approaches that explicitly and exclusively targeted general ER skills were not included in any of the interventions [35], [67], [68].

### Measures

#### Emotion-Regulation Skills Questionnaire

To assess successful ER skills application, we used the German version of the Emotion-Regulation Skills Questionnaire (ERSQ [69]). The ERSQ is a self-report questionnaire consisting of 27 items. Each of the nine skills is assessed on a 5-point Likert-type scale (*0 = not at all* to *4 = almost always*), which are preceded by the stem *“Last week I …”*. Based on the ACE model, successful skills application is assessed through the following nine subscales: *awareness* (e.g., “I paid attention to my feelings.”), *sensations* (e.g., “My physical sensations were a good indication of how I was feeling.”), *clarity* (e.g., “I was clear about what emotions I was experiencing.”), *understanding* (e.g., “I was aware of why I felt the way I felt.”), *modification* (e.g., “I was able to influence my negative feelings.”), *acceptance* (e.g., “I accepted my emotions.”), *tolerance* (e.g., “I felt I could tolerate my negative feelings.”), *readiness to confront distressing situations when necessary to attain personally relevant goals* (e.g., “I did what I had planned, even if it made me feel uncomfortable or anxious.”), and *self-support* (e.g., “I supported myself in emotionally distressing situations.”). The total score of the ERSQ is computed as the mean of all 27 items. Previous studies have provided evidence that all scales of the ERSQ have good internal consistency; at least adequate retest reliability; good convergent, discriminate, and factorial validity; and significant sensitivity to change [33], [57], [58], [62], [69]–[73]. As indicated in Table 1, in the present study, the internal consistencies were very good for the ERSQ score at all four assessment points (*α*T1–4 = .96–.97).

**Table 1:** Descriptive Statistics for BDI and ERSQ over Time.

	Time 1				Time 2				Time 3				Time 4			
*Measure*	*M*	*SD*	*N*	α	*M*	*SD*	*N*	α	*M*	*SD*	*N*	α	*M*	*SD*	*N*	α
ERSQ_Total_	2.87	.74	171	.96	3.07	0.76	112	.97	3.20	0.77	111	.96	3.25	0.79	101	.96
DASS_Stress_	8.12	4.78	172	.88	7.44	4.48	114	.90	6.94	4.57	113	.86	6.72	4.66	105	.91
DASS_Depression_	8.11	5.57	172	.92	7.05	4.82	114	.92	6.31	4.76	113	.91	5.72	4.75	105	.94
DASS_Anxiety_	5.06	4.65	172	.85	4.41	3.72	114	.87	4.41	4.39	113	.86	4.23	4.18	105	.87

*Note.* ERSQ = Emotion-Regulation Skills Questionnaire, DASS = Depression Anxiety Stress Scale, *M* = Mean, *SD* = Standard Deviation, *N* = Number of completers, Means and standard deviations are based on FIML estimation.

#### Depression Anxiety Stress Scale–21-item version

To assess separate scores for DSS, ASS, and GDS, we used the Depression Anxiety Stress Scale (DASS-21 [74]). Sample items include “I couldn’t seem to experience any positive feelings” (depression), “I felt scared without any good reason” (anxiety), and “I found it hard to wind down” (general distress). Participants indicate how much each item applied to them over the past week on a 4-point Likert-scale. All subscales demonstrate very good internal consistency (α ranging from.88 to.94) in a clinical sample [75] and adequate retest-reliability (coefficients ranging from.71 to.81) in a clinical sample [76]. In the current study, the DASS-21 showed good internal consistencies for all four points of assessment (*α*T1–4 = .85–.92).

### Statistical Analyses

#### Cross-sectional association and associations of change

To test whether successful ER skills application is negatively correlated with DSS, ASS, and GDS (Hypothesis 1), we computed Pearson product-moment coefficients (*r*) for all four assessment points. To determine whether changes in successful ER skills application are negatively associated with changes in DSS, ASS, and GDS over the course of treatment for MDD (Hypothesis 2), we used bivariate latent growth curve (LGC) modeling, which is based on structural equation modeling (SEM) [77], [78]. Statistically, SEM represents an extension of general linear modeling procedures, such as ANOVA and multiple regression analysis. However, we prefer SEM to more parsimonious approaches, as SEM 1) allows for complex hypotheses that include interactions and reciprocal relationships to be tested, 2) allows for latent variable modeling to be used and thereby the effects of random measurement error to be corrected, and 3) allows for a model to be tested as an explanation of the underlying process, which has given rise to the empirical data.

**Figure 1:**
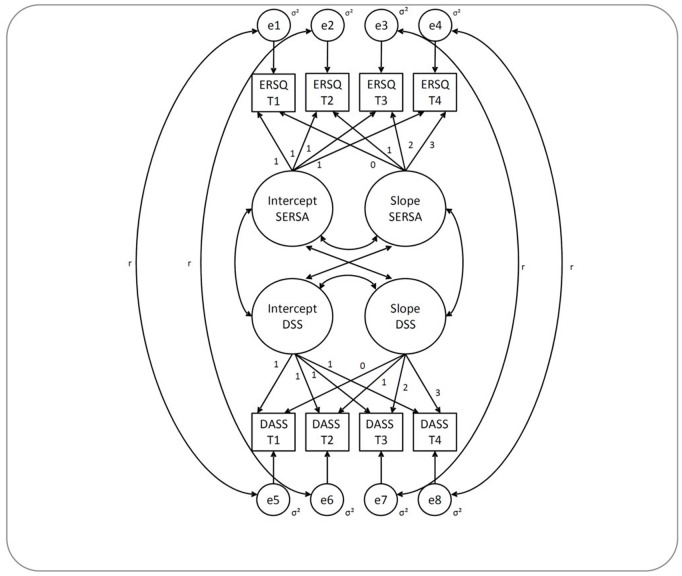
Path Diagram of the Bivariate Growth Curve Model. ERSQ = Emotion-Regulation Skills Questionnaire, DASS = Depression Anxiety Stress Scale, SERSA = Successful Emotion Regulation Skills Application, DSS = Depressive Symptoms Severity, e = residual error, σ^2^ = variance, r = cross-construct error covariance (set equal across time); residual errors were allowed to covary across constructs within time to avoid bias due to variance related to specific assessment occasions and to increase model parsimony [80].

In bivariate LGC, an individual growth curve is calculated for each of the two variables, and each growth curve correlated with the other growth curve. As an example of the association between ER and DSS, Figure 1 illustrates the path diagram of the bivariate latent growth curve model that is used in this study. The means of individual slopes and intercepts describe the group trend of change in the total sample (fixed effects), and the variability of the mean slope and intercept factors (random effects) represent the extent of intraindividual change [79], [80]. The correlations of intercepts indicate the strength of the association between the initial level of successful ER skills application and the initial level of DSS, ASS, and GDS, and the slopes of the correlations indicate the strength of the association between changes in successful ER skills application and changes in depression, anxiety and general distress during treatment [80], [81]. Following procedures proposed by Grimm [80], we fixed loadings for the intercept to one and fixed loadings for the slope to model linear growth, starting with zero for the first assessment point and ending with three for the final measurement at T4.

#### Prediction of subsequent latent change

To test whether successful ER skills application predicts subsequent reduction of DSS, ASS, and GDS (Hypothesis 3), we used latent change score analyses (LCS [78], [82]). LCS models have recently been introduced into treatment outcome research to help identify relevant predictors of change by clarifying reciprocal pathways between two (or more) variables over time (e.g., [83]–[88]). LCS modeling integrates latent growth curve models and cross-lagged regression models to examine reciprocal dynamic processes between two variables. More specifically, time-lagged associations between Variable A and subsequent changes in Variable B and time-lagged associations between Variable B and subsequent changes in Variable A are estimated in the same model. To the extent that the influence of an unknown third variable associated with the dependent variable can be excluded (e.g., through sound theoretical assumptions or statistical procedures), significant associations between Variable A and subsequent changes in Variable B provide evidence for a causal effect of A on B (and vice versa).

**Figure 2:**
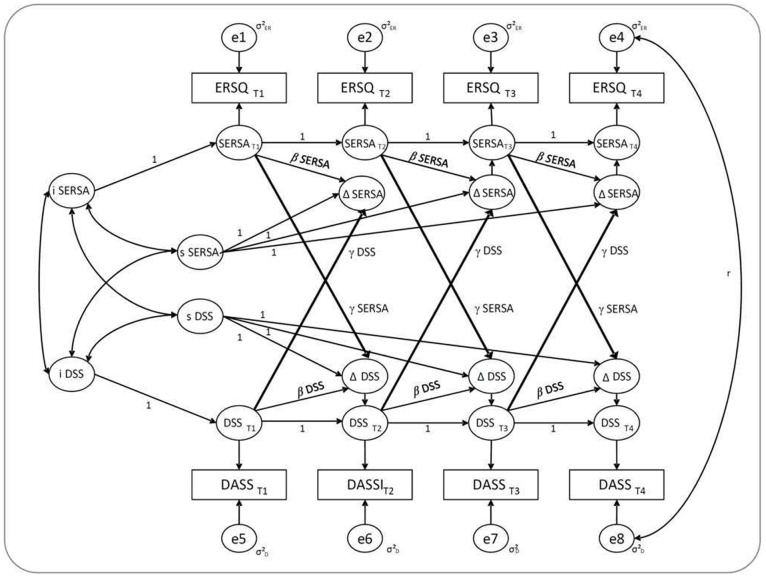
Path Diagram of the Bivariate Latent Change Score Model. ERSQ = Emotion-Regulation Skills Questionnaire, DASS = Depression Anxiety Stress Scale, SERSA = Successful Emotion Regulation Skills Application, DSS = Depressive Symptoms Severity, r = cross-construct error covariance, i = intercept, s = slope, γ = coupling parameter, β = proportion parameter, Δ = latent change score; for purpose of clarity, cross-construct error covariances are only shown for T4, but are also included for the other measurement points; error variances were set equal within constructs; loadings of growth factors and autoregressive proportions were set equal to one; proportion and coupling parameters were set equal across time within constructs; for model identification, means of errors and intercept of observed variables were set equal to zero [81], [89].

Figure 2 illustrates the LCS model that we used to test Hypothesis 3 (as an example, we utilized the association between successful ER skills application (SERSA) and DSS). As indicated in the figure, the trajectory for true scores of both variables comprises an initial level of the unobserved score (intercept) and the accumulation of *true* latent changes in the unobserved variable. Latent change scores (ΔDSS and ΔSERSA in Figure 1) are computed as a function of (a) a constant change factor (slope), referring to systematic change over time; (b) a proportion parameter (β), representing the influence of the same variable at the previous measurement; and (c) a coupling parameter (γ), representing influence of the other variable at the previous time point. These coupling parameters describe dynamic aspects of the model, as they represent the impact of one variable at time *t-1* on the other variable at the next point of time *t* [80], [81].

By setting different restrictions for the coupling parameters, specific hypotheses about the dynamic associations of, e.g., depression and ER skills application can be tested. Following the recommendations of Ferrer and McArdle [81] and McArdle and Grimm [89], we compared the model fit across the unrestricted model and the three nested models resulting from restricting the coupling parameters in accordance with assumptions regarding the prospective associations. In particular, we tested the following models (as an example, we again present the associations between DSS and SERSA): (a) a coupling effect exists for both parameters (bidirectional model, γSERSA ≠ 0, γDSS ≠ 0), (b) a coupling effect exists only for the SERSA to DSS association (unidirectional model, γSERSA ≠ 0, γDSS = 0), (c) a coupling effect exists only for the DSS to SERSA association (unidirectional model, γSERSA = 0, γDSS ≠ 0), and (d) no coupling effects exist for any of the parameters (γSERSA = 0; γDSS = 0). More specifically, we tested whether the bidirectional or either of the unidirectional models would show significantly better fit than the no-coupling model. The same procedure was applied for the LCS analyses for ASS and GDS. The cross-sectional associations between both variables were incorporated by a) allowing the error variance to covary between each point of assessment and b) correlating the intercept of SERSA with the intercept of DSS, ASS, or GDS.

The evaluation of the SEM model fit was based on current recommendations [90], [91]. Aside from the *χ^2^* statistic, we used three fit indices that were most suitable for our data characteristics (moderate sample size and missing values). Hu and Bentler [91] suggested that *good fit* is indicated by Comparative Fit Index (CFI) values greater than or equal to.95, Standardized Root Mean Square Residual (SRMR) values less than or equal to.08, and Root Mean Square Error of Approximation (RMSEA) values less than or equal to.06. We also report the RMSEA confidence interval and the *p* value for the null hypothesis that the RMSEA coefficient in the population is not greater than.05 (*p* close fit [92]).

With regard to missing values, we used the full information maximum likelihood estimation (FIML), which is recommended for use with longitudinal data models [80] and is considered to be superior to other methods, such as listwise or pairwise deletion [93]. Analyses were performed with an alpha level of 0.05, and bidirectional tests were used for all of the hypotheses. We used SPSS 19 (SPSS Inc., Chicago, IL, USA) for the preliminary analyses and MPlus 7 for SEM [94].

## Results

Preliminary analyses confirmed that all the statistical assumptions (normality, linearity, collinearity, and reliability) required for using SEM with FIML were met. Indicators suggested a good (*χ^2^* and RMSEA statistics) to very good (SRMR and CFI values) model fit for the LGC model and a good fit for the LCS model (see Table 2).

**Table 2:** Fit indices of the models tested.

Model	χ^2^	df	χ^2^/df	SRMR	CFI	RMSEA (*p* of close-fit)	90%-CI of RMSEA
Bivariate latent growth curve model							
ERSQ & DASS_Stress_	29.78	21	1.42	.04	.99	.05 (.48)	.00–.09
ERSQ & DASS_Depression_	34.51^*^	21	1.64	.04	.98	.06 (.29)	.02–.10
ERSQ & DASS_Anxiety_	31.63	21	1.51	.04	.99	.05 (.40)	.00–.09
Bivariate latent change score model							
ERSQ & DASS_Stress_	25.50	23	1.11	.03	.99	.03 (.79)	.00–.07
ERSQ & DASS_Depression_	24.74	23	1.08	.03	.99	.02 (.81)	.00–.07
ERSQ & DASS_Anxiety_	29.36	23	1.28	.05	.99	.04 (.63)	.00–.08

*Note.* IFI = Incremental Fit Index; CFI = Comparative Fit Index; RMSEA = Root Mean Square Error of Approximation; CI = Confidence Interval; ERSQ = Emotion-Regulation Skills Questionnaire; BDI = Beck Depression Inventory; ^**^ *p*<.01.

### Cross Sectional Associations and Associations of Change

As shown in Table 3, the ERSQ total score was negatively correlated with DSS, ASS, and GDS at all four assessment points. This finding supports the hypothesis that successful ER skills application is negatively associated with depressive and anxiety symptom severity as well as psychological distress. According to Cohen [95], the correlation coefficient indicates a moderate to large effect size.

**Table 3:** Correlations of ERSQ_total_ Score and the DASS Scales for Each Assessment Point.

Measure	r Time 1	r Time 2	r Time 3	r Time 4	r Total Time
DASS_Stress_	−.48^**^	−.55^**^	−.46^**^	−.60^**^	−.52
DASS_Depression_	−.62^**^	−.62^**^	−.61^**^	−.68^**^	−.63
DASS_Anxiety_	−.45^**^	−.57^**^	−.49^*^	−.53^**^	−.51

*Note.* ERSQ = Emotion-Regulation Skills Questionnaire, DASS = Depression Anxiety Stress Scale, *r* = Pearson-Correlation, *r* Total Time* = *correlation averaged across all assessment points; ^*^ *p*<.05, ^**^p<.01.

Bivariate LGC modeling was conducted to examine whether changes in successful ER skills application are associated with changes in DSS, ASS, and GDS. The mean intercepts and slopes for successful ER skills application, DSS, ASS, and GDS significantly differed from zero (see Table 4), suggesting that significant intraindividual change in these variables occurred in the sample (fixed effects). Moreover, for all the variables (except for the anxiety scale of the DASS-21), the variance of the mean intercept and slope significantly differed from zero, suggesting that the intraindividual change significantly differed across individuals (random effects). For successful ER skills application, DSS, ASS, and GDS, the correlations between the intercepts and slopes were nonsignificant, indicating that a change in the variables is not associated with the pretreatment level. As also shown in Table 4, the intercept of the ERSQ was significantly (negatively) correlated with the DSS, ASS, and GDS intercepts. This finding replicates the findings from the cross-sectional correlations on a latent level; that is, higher pretreatment successful ER values indicate lower pretreatment symptom severity.

**Table 4:** Latent Growth Curve Model: Parameter Estimates for Intercepts, Slopes and Correlations of Slopes and Intercepts.

Measure	Intercept				Slope				Correlation of Slopes		Correlation of Intercepts	
	*M*	*SE*	*σ^2^*	*SE*	*M*	*SE*	*σ^2^*	*SE*	*Estimate*	*SE*	*Estimate*	*SE*
DASS_Stress_	8.03^***^	.35	15.25^***^	2.38	-.48^***^	.11	.64^*^	.29	-.061^*^	.029	−1.53^***^	.288
ERSQ_total_	2.90^***^	.06	.43^***^	.06	.12^**^	. 02	.02^**^	.01	–	–	–	–
DASS_Depression_	7.99^***^	.40	21.67^***^	3.15	-.82^***^	.12	.76^***^	.35	-.094^**^	.034	−2.24^***^	.348
ERSQ_total_	2.90^***^	.06	.44^***^	.06	.13^**^	.02	.02^*^	.01	–	–	–	–
DASS_Anxiety_	4.85^***^	.32	13.76^***^	2.06	-.25^**^	.10	.17	.22	.005	.023	−1.31^***^	.265
ERSQ_total_	2.90^***^	.06	.04^***^	.06	.13^***^	.02	.02^*^	.01	–	–	–	–

*Note.* Unstandardized parameter estimates are presented; *M* = Mean, *SE* = Standard Error, *σ^2^* = Variance, ERSQ = Emotion Regulation Skills Questionnaire, DASS = Depression Anxiety Stress Scale; Residual variance set equal over time within construct; ^*^ *p*<.05, ^**^p<.01, ^***^ *p*<.001.

Finally, consistent with Hypothesis 2, the slopes of successful ER skills application and DSS and the slopes of successful ER skills application and GDS were significantly (negatively) correlated. This finding indicates that an increase in overall successful ER skills application is significantly associated with a decrease in DSS and GDS and vice versa. However, a significant correlation was not found for the slopes of successful ER and ASS.

### Prediction of the Subsequent Latent Change

Time-lagged associations between successful ER skills application and DSS, ASS, and GDS were tested by using the LCS model illustrated in Figure 2. As shown in Table 5, the cross-lagged effect from successful ER skills application on subsequent changes in DSS (γSERSA = −2.65, *p* = .035) was significant and negative. Consistent with Hypothesis 3, this finding indicates that successful ER skills application predicts changes in the subsequent level of DSS. Patients reporting more successful ER skills application were likely to experience a greater reduction of DSS. In contrast, the other coupling effect predicting changes in successful ER skills application from previous DSS scores was nonsignificant (γDSS = -.01, *p* = .95). These findings are consistent with those of a previous study by Radkovsky and colleagues62 that assessed the cross-sectional and bivariate relationship between ER and DSS measured by the BDI. The cross-lagged effect from successful ER skills application on subsequent changes in GDS (γSERSA = −1.12, *p* = .23) and ASS (γSERSA = -.20, *p* = .76) was negative but nonsignificant. Additionally, the other coupling effects predicting changes in successful ER skills application from previous GDS (γstS = -.03, *p* = .21) and ASS (γASS = -.02, *p* = .52) scores were nonsignificant.

**Table 5:** Bivariate Latent Change Score Model: Estimates of Regression Coefficients.

	Coupling Parameter				Proportion Parameter			
	γ_SERSA_ (SERSA → _Δ_StS)		γ_StS_ (StS → _Δ_SERSA)		β_SERSA_ (SERSA → _Δ_SERSA)		β_StS_ (StS → _Δ_StS)	
Model	Estimate	S.E.	Estimate	S.E.	Estimate	S.E.	Estimate	S.E.
DASS_Stress_	−1.13	0.93	−.03	.03	−.55^***^	.13	−.74^***^	.15
DASS_Depression_	−2.65^*^	1.25	−.01	.03	−.46^**^	.17	−.79^***^	.17
DASS_Anxiety_	−0.20	0.65	−.02	.03	−.47^***^	.12	−.79^***^	.18

*Note.* Unstandardized parameter estimates are presented; coefficients β_SERSA_, β_SS_, γ_SERSA_ and _DSS_ as denoted in Fig. 2; SE = Standard Error, SERSA = successful Emotion Regulation skills application, StS = Symptoms Severity, _Δ_ = Difference between two subsequent time points of assessment; ^*^ *p*<.05, ^**^p<.01, ^***^ *p*<.001.

For each of the three LCS model, we tested whether the fit of the bidirectional model (e.g., unrestricted estimation of γSERSA and γDSS) and the two unidirectional models (γSERSA or γDSS set to zero) differed significantly from the fit of the no-coupling model (γSERSA and γDSS set to zero). For the LCS models, neither the ASS nor the GDS model resulted in a significant improvement in the model fit relative to the no-coupling model. However, we found a significant improvement for the unidirectional model relative to the no-coupling model in the DSS model (see Table 6). This finding again indicates that changes in general ER predict subsequent changes in DSS but not vice versa.

**Table 6:** Bivariate LCS Models: Stepwise Test of Coupling Effects (Δ χ^2^/Δ df for comparisons with no-coupling model).

	Bidirectional model	Unidirectional model γ_SERSA_	Unidirectional model γ_StS_
	SERSA ↔ StS	SERSA →_Δ_StS	StS →_Δ_SERSA
	γ_SERSA_ ≠ 0, γ_StS_ ≠ 0	γ_SERSA_ ≠ 0, γ_StS_ = 0	γ_SERSA_ = 0, γ_StS_ ≠ 0
DASS_Stress_	4.9/2	3.3/1	3.4/1
DASS_Depression_	4.7/2	4.6/1^*^	0.4/1
DASS_Anxiety_	0.6/2	0.1/1	0.5/1

*Note.* Coefficients γ_SERSA_ and γ_SS_ as denoted in Fig. 2; SERSA = Successful Emotion Regulation Skills Application, StS = Symptoms Severity; ^*^ *p*<.05.

## Discussion

Research suggests that deficits in emotion regulation (ER) are an important factor driving the maintenance of depression and hence a promising treatment target when working to reduce depressive symptoms. A particular advantage of enhancing individuals’ *general* ER skills (i.e., skills that are effective for coping with a broad range of undesired affective states) is that these skills can also be assumed to facilitate individuals’ ability to cope with symptoms that often co-occur with depressive symptoms and that arguably contribute to the maintenance of depression, such as anxiety symptoms. However, at this point, the associations between the successful application of adaptive ER skills and symptoms of depression and anxiety have not been investigated during the course of treatment in individuals who meet the criteria for MDD. Therefore, we assessed self-reports of successful ER skills application, depressive symptom severity (DSS), and anxiety symptom severity (ASS) over four assessment points in a sample of 175 inpatients receiving treatment with cognitive behavioral therapy for MDD. To assess the potentially confounding effects of general distress as a common factor of depression and anxiety, we also assessed self-reports of general distress severity (GDS). After testing whether ER is cross-sectionally associated with DSS, ASS, and/or GDS, we used latent curve modeling to test whether changes in successful ER skills application are negatively associated with changes in DSS, ASS, and/or GDS, and we used latent change score modeling to test whether successful ER skills application predicts the subsequent reduction of DSS, ASS, and/or GDS. In the results, successful ER skills application was significantly associated with (lower levels of) DSS, ASS, and GDS. Moreover, an increase in successful ER skills application was significantly associated with a decrease in DSS and GDS but not ASS. Finally, successful skills application significantly predicted the subsequent reduction of DSS. Unexpectedly, successful ER skills application significantly predicted the subsequent reduction of neither ASS nor DSS.

The finding that successful ER skills application was consistently associated with lower levels of (subsequent) DSS contributes to a body of evidence supporting the hypotheses that deficits in ER constitute an important factor driving the risk for and the maintenance of MDD [21], [35], [96]–[98]. Additionally, the findings that successful ER skills application was cross-sectionally associated with lower levels of ASS and that successful ER skills application was negatively associated with ASS provide some support for the transdiagnostic importance of general ER skills [23], [28], [99], [100]. However, the findings that (a) the cross-sectional associations were smaller for ASS than for DSS, (b) the association between the slope for successful ER skills application and the slope for ASS was nonsignificant, and (c) successful ER skills application did not significantly predict subsequent ASS indicates that general ER skills might be more important in the context of depression than in the context of anxiety.

This hypothesis is consistent with preliminary evidence indicating that depressed individuals often struggle with a broad range of aversive affective states, including sadness, dysphoria, helplessness, hopelessness, a lack of positive emotions, stress/tension, anxiety, guilt, and shame [101], [102], whereas in the context of anxiety disorders, the focus is commonly more restricted to an external threat, anxiety, or its somatic symptoms [101], [103]–[105]. Thus, in the context of depression, general ER skills should be particularly helpful, as they help an individual to cope with a broad range of negative affective states that he or she is suffering from. In contrast, the ability to cope with anxiety-related problems might instead be facilitated by enhancing specific ER skills that are particularly effective for coping with anxiety. Moreover, deficits in ER are associated with difficulties in sustaining and/or increasing positive emotions. As anhedonia is one symptom thought to separate depression from anxiety disorders, the enhancement of general emotion regulation skills should be particularly effective for patients suffering from depression.

Another interesting finding in the present study is that successful ER skills application was not significantly associated with general distress in the LCS analyses. This finding is consistent with multiphase theories of affect generation [35], [106]–[108]. These theories postulate that a perceived misfit between salient goals and needs and the perception of goal attainment and need satisfaction initially elicits unspecific negative affect; furthermore, individuals subsequently analyze the misfit between the perceived state and the desired state in detail and make prognoses about how the misfit will likely develop in the future. For these cognitive processes, the perceived ability to cope with one’s emotions is crucial. If the individual anticipates that she/he will be able to cope with the undesired affective state, no challenging secondary affective reactions, such as anxiety or depression, will occur. However, if the individual anticipates having trouble coping with the undesired affective state, anxiety will occur. If the individual anticipates that he/she will be completely unable to control the undesired affective state and therefore assumes that this state will be stable over time, depressogenic schema will be activated [109]. The findings from the present study provide support for these theories in a clinical context, as they suggest that the ability to cope with general distress does not necessarily reduce the distress itself but may prevent the development of symptoms of a mental disorder (such as depression).

Strengths of this study include the use of a large and carefully diagnosed clinical sample, multiple assessments, a routine health care setting, and advanced statistical methods that have recently been introduced to clarify reciprocal associations in longitudinal data. A major limitation of the study includes the exclusive use of self-reports, as the process of emotion regulation notably functions in an explicit and implicit manner. Therefore, the exclusive use of self-reports may miss crucial information regarding the participants’ implicit emotion regulation strategies, as the participants may not be fully aware of these strategies. Thus, future research should use multiple methods, e.g., experimental [110] and biological [111] paradigms, to assess ER in treated and untreated samples while combining latent change score analysis with multitrait-multimethod approaches [112] to diminish the occurrence of measurement errors. Moreover, the ER measure that was used in the present study assesses how participants respond to their “negative feelings” or “emotions” and hence does not discriminate between ER skills across distinct affective states. This is problematic because the measure does not help to identify which skills are particularly helpful for coping with a specific form of psychopathology. Thus, future research should assess regulation skills separately across various affective states [113] and should test the hypothesis that successfully utilizing arguably effective ER skills to cope with several undesired affective states is particularly important in the context of depression, whereas in the context of anxiety, the ability to effectively use anxiety regulation skills might be far more important than the ability to effectively use regulation skills related to other affective states.

Moreover, adaptive ER notably needs to be conceptualized as a dynamic process in which multiple strategies are used [19], [34]. Thus, future research should assess how affective and regulatory processes interact over time and which patterns of ER strategy utilization are most effective for coping with specific mental-health problems.

An additional limitation of this study is the selection criteria, which limit the generalizability of the results, as only German-speaking inpatients with a current diagnosis of MDD and a pre-treatment BDI score of 11 or above were eligible for the study. Moreover, because of the selection criteria, the DASS-21 was merely approximately normally distributed at certain assessment points. However, nonparametric tests (e.g., Spearman’s Rho) achieved comparable results. In addition, future studies 1) should prolong the interval of assessment, as the first three weeks of inpatient treatment may be too short to assess substantial effect of emotion regulation on depression, anxiety, and distress, and 2) should use a larger sample size owing to the complexity of the model.

Finally, significant associations might be driven by a third factor that affects both ER and symptoms of mental disorders. As the number of factors that can be assessed and statistically controlled for is always limited, future research should focus on experimental studies (i.e., randomized clinical trials) in which the focus on enhancing either general or specific ER skills is systemically varied. If this research assesses how the type of psychopathology moderates the effects of such interventions on psychopathology, the extent to which enhancing general ER skills can be used within transdiagnostic interventions to effectively foster patients’ ability to cope with various challenges for mental health can be clarified.

## Supporting Information

Demographic Data S1 (SAV)

Data S1 (SAV)
